# HSV-2 Vaccine: Current Status and Insight into Factors for Developing an Efficient Vaccine

**DOI:** 10.3390/v6020371

**Published:** 2014-01-24

**Authors:** Xiao-Peng Zhu, Zaka S. Muhammad, Jian-Guang Wang, Wu Lin, Shi-Kun Guo, Wei Zhang

**Affiliations:** 1 The 2nd Clinical Medical College, Wenzhou Medical University, Wenzhou 325025, Zhejiang, China; E-Mails: zhuxp200@foxmail.com (X.-P.Z.); linwu05linwu@gmail.com (W.L.); zhuangshikun@126.com (S.-K.G.); 2 School of International Studies, Wenzhou Medical University, Wenzhou 325025, Zhejiang, China; E-Mail: muhammad.zaka@hotmail.com; 3 School of Basic Medical Sciences, Wenzhou Medical University, Wenzhou 325025, Zhejiang, China; E-Mail: wjg@wzmc.net

**Keywords:** herpes simplex virus 2, vaccine, mucosal immunity, delivery route, adjuvant, sex hormone

## Abstract

Herpes simplex virus type 2 (HSV-2), a globally sexually transmitted virus, and also one of the main causes of genital ulcer diseases, increases susceptibility to HIV-1. Effective vaccines to prevent HSV-2 infection are not yet available, but are currently being developed. To facilitate this process, the latest progress in development of these vaccines is reviewed in this paper. A summary of the most promising HSV-2 vaccines tested in animals in the last five years is presented, including the main factors, and new ideas for developing an effective vaccine from animal experiments and human clinical trials. Experimental results indicate that future HSV-2 vaccines may depend on a strategy that targets mucosal immunity. Furthermore, estradiol, which increases the effectiveness of vaccines, may be considered as an adjuvant. Therefore, this review is expected to provide possible strategies for development of future HSV-2 vaccines.

## 1. Introduction

**Figure 1:**
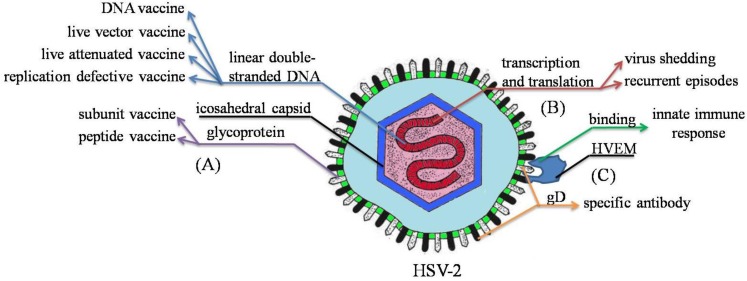
Illustration of pathogenesis and immune responses of HSV-2 in vaccine development. (**A**) HSV-2 glycoproteins, especially gB and gD, are widely used to develop subunit vaccine and peptide vaccine. DNA has also been frequently used for vaccine development; (**B**) The transcription and translation of the genes lead to HSV-2 shedding and recurrent episodes; (**C**) The ability of gD to induce specific antibody is the strongest. The combination of gD with HVEM triggers immediate innate immune response, which leads to subsequent adaptive immune response.

Herpes simplex virus type 2 (HSV-2), a linear double-stranded DNA virus of the *Herpesviridae* family and a major cause of genital ulcer diseases, is transmitted by sexual contact or via the maternal-neonatal relationship. Despite multiple measures adopted to control the diseases, this virus still infects at least 500 million people around the world [1]. HSV-2 reaches a latent state in the sensory nerve root ganglia and reactivates when the immune function of the body declines, causing recurrent episodes (Figure 1) [2]. The rapidly cleared episodes of HSV shedding are present in HIV co-infected persons [3]. However, the mechanisms of the latency are still unknown. Recent studies show that HSV-2 increases the risk of HIV-1 acquisition [4,5,6]. The potential biological mechanisms by which HSV-2 increases risk of HIV-1 infection include disruption of the genital epithelium, recruiting activated target cells for HIV-1, decreasing innate mucosal immunity and inducing a mucosal inflammatory response [7]. HSV-2-infected monocyte-derived dendritic cells (moDCs) increase retinoic acid production and high α4β7 expression on CD4^+^ T cell, which can be spotted by HIV-1 [8]. HSV-2 can also damage the protective function of mucosal Langerhans cells (LCs) through abrogating the function of langerin, which enhances the susceptibility of HIV-1 [9].

The most effective and economical way to overcome HSV-2 is to develop a vaccine. With much work done towards this end, great progress has been made in the development of an HSV-2 vaccine in the past several decades [10]. Nevertheless, no ideal vaccine is currently available [11]. In order to facilitate the discovery process of effective vaccines against HSV-2, this review analyzes the key factors of developing effective vaccines and the latest progress in HSV-2 vaccine under the categories of HSV-2 pathogenesis, immune response to HSV-2, vaccine formulation, route of immunization, adjuvant and influence of sex hormones.

## 2. HSV-2 Pathogenesis

Though a great advance has been made in the study of HSV-2 pathology recently, little is known about the pathogenesis, which needs to be further examined [2]. HSV-2 entry requires the combination of viral glycoprotein D (gD) with its receptors, including herpesvirus entry mediator (HVEM), nectin-1 and -2, and specific sites in heparan sulfate [12]. The interaction between gD and HVEM during acute infection with HSV decreases the subsequent CD8^+^ recall response at the genital mucosa [13]. The interaction also leads to the weakening in the regulation of HVEM surface expression and alters early innate immune response against infection in mice [14,15]. HSV-2 alters innate immune responses by decreasing the level of type I interferon (IFN-α and IFN-β), increasing the level of type II interferon (IFN-γ) [16], and decreasing production of secretory leukocyte protease inhibitor (SLPI) [17]; such a process causes immune evasion. Recent studies reveal that HSV-2 blocks dendritic cell (DC) maturation, induces DC apoptosis, and triggers the release of proinflammatory cytokines [18,19], which increases HIV-1 susceptibility. HSV-2 reactivation leads to recurrent episodes ranging from mild to severe cases [2]. In terms of the frequency of recurrent episodes, seropositive individuals are divided into two groups: symptomatic individuals and asymptomatic individuals [20]. However, it remains unknown why frequency and severity of recurrent diseases are different among these individuals. Recent findings show that symptomatic individuals and asymptomatic individuals differ in the levels of HSV-specific T cell repertoires and T cell response to HSV epitopes [20,21,22,23,24].

## 3. Immune Response to HSV-2

### 3.1. Innate Immune Response: An Immediate Nonspecific Protection

A powerful and robust immune response to HSV-2 requires both the innate immune response and the adaptive immune response. Most studies deal with adaptive immune response due to its function for viral clearance and generation of long-term memory. However, some recent studies provide the findings that the innate immune response induces immediate nonspecific protection against HSV-2, indicating its important role in response to HSV-2 [25].

The interaction between virus and innate immune cells initiates the innate immune response via pattern recognition receptors (PRR) that recognize pathogen-associated molecular patterns (PAMP) like viral DNA and RNA. The major PRRs, Toll-like receptors (TLR), are expressed by innate immune cells inclusive of mononuclear phagocytes, dendritic cells (DC) and NK T cells. The function of TLRs, such as TLR9 [26], TLR3 [27] and TLR2 [28], has been demonstrated to induce the immune response. In a recent study, ligand for TLR5 inhibited HSV-2 replication in genital epithelial cells, indicating that TLR5 may be associated with the immune response against HSV-2 [29]. After viral recognition and TLR activation, immune cells produce type I interferon (IFN), resulting in the antiviral state. The antiviral state is achieved through the activation of RNA-dependent protein kinase (PKR) by the IFN-α1 transgene, which is a major pathway to induce the antiviral state [30]. The resultant antivirus state in the vagina can be regulated by four major antigen-presenting cell (APC) subsets including langerhans cells (LCs), CD14^−^ lamina propria-dendritic cells (LP-DCs), CD14^+^ LP-DCs and macrophages polarize CD4^+^ T cells, which elicit Th1, Th2 and Th22 responses by inducing theexpression of migration receptors [31].

#### Cytokines: A Blessing or a Curse

Cytokines can cause positive or negative effects on immune responses in terms of the type they belong to. For example, Type I IFNs, widely investigated, play an important role in resistance to genital HSV-2 infection [32,33]. IFN-λ, a recent discovery, also shows potential ability to resist HSV-2 infection [34]. Besides, IL-15, important for TLR ligand-induced immune responses [35,36], acts independently of NK/NKT cells in mediating the innate response [37]. Further studies suggest that ligands for TLR-3 and TLR-9 induce no protection against HSV-2 in the absence of IL-15 *in vivo* [38]. In addition, the anti-HSV-2 function of plasmacytoid DCs (pDCs) and NK cells has been demonstrated [26,33,39]. However, TNF-α antibody reduced mortality caused by HSV-2 infection in CXCL10 deficient mice, which indicated that TNF-α works as a principal mediator of mortality against HSV-2 [40].

### 3.2. Adaptive Immune Response

The adaptive immune response, triggered by the innate immune response, consists of cellular and humoral immunity. The main effect of the adaptive immune response is eliminating the virus and inducing long-term memory.

#### 3.2.1. Cellular Immunity: Keeping a Balance between CD4^+^ T Cells and CD8^+^ T Cells

Both CD4^+^ T cells and CD8^+^ T cells are required in the HSV-2 specific immune response. Early studies showed the significance of CD4^+^ T cells in anti-HSV-2 immune response [33]. Compared with CD4^+^ T cells, CD8^+^ T cells persisted in genital skin and mucosa at the dermal–epidermal junction (DEJ), the portal of the neuronal release of reactivating virus for a prolonged period after herpes lesions cleared [41]. Data from subsequent studies indicate that DEJ CD8aa^+^ T cells (the dominant resident population of DEJ CD8^+^ T cells), tissue-resident cells, may play a role in immune surveillance and in initial containment of HSV-2 reactivation in human peripheral tissue [42]. To be more specific, memory CD8^+^ T cells, expressions of tissue-homing integrins CD103 and VLA-1 in the genital tract, eliminate virus and control herpes virus reactivation and latency [43]. Memory CD8^+^ T cells produced IFN-γ while memory CD4^+^ T cells produced both IFN-γ and TNF-α when stimulated with HSV antigens *in vitro* [43]. Recently, a new HSV-2 vaccine strategy based on tissue-resident memory T cells has been established. This strategy consists of two steps: parenteral vaccination to induce systemic T cell responses, followed by recruitment of activated T cells via topical chemokine application to the restrictive genital tract [44]. This approach reduced the spread of HSV-2 into the sensory neurons and prevented the development of clinical disease in mice, and is likely to be a potential alternative in future vaccine development [44].

Regulatory T cells (Tregs), known to inhibit the immune response, play a new role in HSV-2 infection. In some studies, interferon, detected in the draining lymph nodes (dLNs) in Treg-deprived mice, decreased at the infection site. In addition, the migration of natural killer cells, dendritic cells, and T cells to the infection site was delayed, and the proinflammatory chemokine levels in the dLNs were increased, suggesting that Tregs facilitate early protective responses to local viral infection by permitting immune cells into infected tissue [45].

#### 3.2.2. Humoral Immunity: A New Role of B Cells and Secreted Antibody

There is controversy over the function of B cells and the production of IgG and IgA against HSV-2. According to the findings of many studies, B cells were dispensable against HSV-2 infection [33]. Furthermore, some human clinical trials also showed that the vaccine was invalid though it induced significant HSV-2-specific antibodies [46,47]. On the other hand, another experimental result indicated that antibodies induced by the HSV-2 vaccine were associated with protection against HSV-1 [47]. In addition, B cells triggered CD4^+^ T cells to produce IFN-γ in the presence of dendritic cells [48]. It was also demonstrated that pre-challenge levels of pan-HSV-2 IgG (the IgG antibodies response to all of HSV-2 antigens) quantitatively correlated with reduction in HSV-2 challenge virus shedding and increased survival rate following HSV-2 challenge [49]. Transfer of IgG from serum to female genital tract is via neonatal Fc receptor (FcRn), which is expressed in female genital tract epithelial cells and binds IgG in a pH-dependent manner [50,51]. An interesting immunization strategy that consists of fusing gD2 to an IgG Fc fragment induced efficient mucosal and systemic immune response, and provided long-term protection against HSV-2 [52]. A recent study found that HSV specific IgG, though not protective for HSV disease, was the major determinant of viral inhibition in cerebrospinal fluid (CSF), preventing virus recovery in cell cultures [53]. These data point to the importance of B cells and antibodies in protection against HSV-2. Therefore, more research needs to be done in regard to the new function of the two types of humoral immunity.

## 4. Vaccine Formulation

In terms of vaccine components and mechanisms, HSV-2 vaccines are divided as follows: inactivated vaccine, live attenuated vaccine, replication defective vaccine, subunit vaccine, peptide vaccine, live vector vaccine and DNA vaccine. Inactivated vaccines, poorly immunogenic and of low efficacy with the potential to increase susceptibility of cancer, have fallen into neglect.

Live attenuated vaccines, which are non-pathogenic or of low pathogenicity via deleting related genes of virulence, latency or resurrection, have the potential to induce effective protection against HSV-2. In early 2000, a few HSV-2 live attenuated vaccine trials demonstrated that the vaccine only prevented 37.5% of the patients from recurrence [54]. Recently, several HSV-2 ICP0^−^ mutant viruses have been constructed and evaluated for safety and immunogenicity [55]. Among them, HSV-2 0ΔNLS has an optimal balance between avirulence and immunogenicity in mice [55]. Another study reported that 114 of 115 mice immunized with HSV-2 0ΔNLS survived in virus challenge, while 3 of 45 mice immunized with gD2 subunit vaccine survived [56]. In addition, 0ΔNLS immunized mice shed an average 125-fold less HSV-2 MS (wide type) challenge virus per vagina than gD2 immunized mice [56]. These results indicate that the HSV-2 ICP0^−^ induces 10 to 100 times greater protection against genital herpes than the gD2 subunit vaccine, which is a potent HSV-2 vaccine candidate for further study. Another attractive candidate for live attenuated HSV-2 vaccine is HSV 2-gD27 (point mutations of amino acids 215, 222 and 223), which loses its ability for interaction with nectin-1. As is shown in other studies, HSV2-gD27 lost the capacity to infect cells expressing only nectin-1, including neuronal cell lines, and did not infect ganglia in mice [57]. However, the disadvantage of these vaccines is the possibility of reverting to the wild type phenotype.

Replication defective vaccines in which replication related genes are deleted, also exhibit great safety and immunogenicity in HSV-2 vaccine development. Although a disabled infectious single cycle (DISC) mutant with a deletion in the gH gene has no clinical or virologic benefit in the amelioration of genital HSV-2 disease among immunocompetent men and women [58], the new replication defective vaccine candidates, such as ACAM529, CJ2-gD2 and gE2-del virus, have great efficacy against HSV-2 (Table 1).

Subunit vaccines are the most studied HSV-2 vaccines. From the 1970s to 1990s, several subunit vaccine trials had been conducted but failed to provide protection against HSV-2 [39]. So far, the majority of the trials have focused on prophylactic subunit vaccines, mostly using the HSV-2 surface glycoproteins gD and gB. While gD2 is the entry receptor of HSV-2, gB2 is a transmembrane envelope glycoprotein that contributes to the penetration of virions into host cells. A gD2 subunit vaccine with an alum/MPL adjuvant, even if significantly reducing HSV-2 disease in a subgroup analysis of HSV-1- and HSV-2-seronegative women, had no efficacy against genital herpes in men and HSV-1-seropositive women [59]. However, the latest randomized, double-blind efficacy field trial with its focus on the subgroup involving 8,323 women, produced the result that this gD2 subunit vaccine was effective in preventing HSV-1 genital disease and infection but not in preventing HSV-2 disease or infection [47]. The findings of these studies suggest that a simple glycoprotein or several glycoproteins may not have the ability to induce robust immune responses against HSV-2. Fortunately, some new potential subunit vaccines bring new hopes to develop an effective vaccine (Table 1).

Peptide vaccines are synthetic peptides that induce protective immune responses against HSV-2. The peptides mostly target T cell epitopes or B cell epitopes, which have been shown to be protective against HSV-2. Results of recent studies indicate that the HerpV vaccine is immunogenic, generating CD4^+^ and CD8^+^ T cell responses in mice and in HSV-2 positive human participants, providing an indication of a potential vaccine candidate [60,61]. In addition, it was found in the studies that T cells from symptomatic and asymptomatic men and women recognized different herpes epitopes. That is, T cell responses targeting herpes epitopes in symptomatic individuals lead to pathological conditions, while T cell responses targeting herpes epitopes in asymptomatic individuals lead to a protective state [62], promising to develop an effective, “asymptomatic” T-cell epitope-based therapeutic HSV vaccine. Further studies found 4 ID-A-Ags (immunodominant “asymptomatic” antigens) and 1 ID-S-Ag (immunodominant “symptomatic” antigens) by probing whole-ORFome microarrays [62].

By comparison, live vector vaccines that express HSV-2 antigens via heterologous expression vectors like adenovirus and vaccinia virus, also have the capability to trigger a strong immune response against HSV-2. Studies show that the modified vaccinia virus Ankara (MVA) vectors expressing HSV-2 gD induce strong cellular and humoral immunity [63]. In addition, recombinant *Vibrio cholerae* ghosts (rVCG) expressing chlamydial MOMP and HSV-2 gD induce high levels of antibodies against both chlamydial and HSV-2, and trigger a strong Th1 immune response [64]. The major disadvantage of this kind of vaccine is that antibodies against heterologous vectors may exist in the human body, which will influence efficacy of vaccines. Furthermore, the safety of vector vaccines, which may pose a threat to human health, should also be considered.

DNA vaccines, which originated in the 1990s, open up a new path for HSV-2 vaccine development. The gD2 and gB2 genes are widely used to construct HSV-2 DNA vaccines because these two types of genes are the main antigens to induce immune response. Recently, a gD2 DNA vaccine has been tested in a double-blind, vehicle-controlled, dose escalation safety and immunogenicity trial. Results show that the vaccine is safe and well tolerated, inducing a specific cellular immune response [65]. Furthermore, some new DNA vaccines, in recent studies, possess the protective potential against HSV-2 (Table 1).

**Table 1:** The most promising HSV-2 vaccines tested in animals in the last five years.

Type	Formation	Adjuvant	Delivery route	Efficacy
Subunit vaccine [66]	gD2ΔTMR340-363 and ICP4383-766	Matrix M-2	s.c. (Scruff of the neck)	Induce humoral and cellular immune responses, reduce recurrent disease
Subunit vaccine [67]	mature form of gG2	CpG	s.c. followed by i.n.	Low disease scores, survival rate: 73%, no neutralization capacity
Subunit vaccine [52]	gD-Fc fusion protein	CpG	i.n.	Induce strong mucosal and systematic immune responses, protection lasts for 6 months
Subunit vaccine [68]	gB, gD, or gB and gD	CpG	i.m. (tibialis anterior muscle)	Induce neutralizing antibody response and T cell response
Subunit vaccine [69]	gC2 and gD2	CpG and alum	i.m. (calf muscle)	Induce neutralizing antibody response and CD4^+^ T cell response, better protection than gC2 or gD2 alone
Subunit vaccine [70]	liposome containing gD21-306-HD, phospholipid, and cholesterol	MPL	s.c.	Induce the level of IFN-γ, reduce disease burden, survival rate: 71%
Replication defective vaccine [71,72]	dl5-29	-	s.c. (Scruff of the neck) [71] or i.m. [72]	Immunogenic and efficacious *in vivo* [71], greater immunogenicity and protection [72]
Replication defective vaccine [73]	ICP0^−^ mutant-based dominant-negative recombinant virus	-	s.c. (left rear flank)	Safe, induce strong HSV-2-specific memory CD4^+^ and CD8^+^ Tcell responses
Replication defective vaccine [74]	ICP8- virus encoded B7-2	-	s.c. (hind flank)	Induce more IFN-γ-producing CD4^+^ T cells, reduce diseases, suppress infection
Live attenuated vaccine/Replication defective vaccine [75]	gE2-del virus	-	Safety test: i.m. (gastrocnemius muscle) or i.v. (tail vein) or ivag immune test: i.m.	Safe, induce incomplete protection
Live attenuated vaccine [55,56]	ICP0^−^ mutant virus	-	Ocular [55], s.c. (right rear footpads) [56]	Safe [55], 10–100 times better protection than gB subunit vaccine, survival rate: 99.1% [56]
DNA vaccine [76]	gD2 plasmid DNA encoding UL46 and UL47	Vaxfectin	i.m. (rear leg)	Induce complete protection, reduce virus reactivation
DNA vaccine [77]	gD2 plasmid DNA	Vaxfectin	i.m.	Induce IgG, reduce virus copies in DRG, survival rate: 80%
DNA vaccine [78]	gD2 and gB2 CTL epitope plasmid DNA	-	i.m. (quadriceps muscle)	Induce serum IgG and Th1 immune response, survival rate: 90%
Inactivated vaccine [79]	whole formalin-inactivated virus	MPL and alum	i.m.	Provide nearly complete protection after challenge
Peptide vaccine [80]	gB T cell and B cell epitope-based peptides	-	i.m. and i.p.	T cell epitopes increase IFN-γ-producing CD8^+^ T cells, B cell epitopes induce high humoral response
Peptide vaccine [60]	32 HSV-2 peptides with HSP70	QS-21 saponin	i.d.	Induce cellular immune response, survival rate: 54%
Peptide vaccine [81]	gB CD8^+^ T cell epitope-based peptides extended by palmitic acid moiety	Self-adjuvant	ivag	Induce specific memory CD8^+^ cytotoxic T cells

s.c., subcutaneous; i.m., intramuscular; i.p., intraperitoneal; i.n., intranasal; i.v., intravenous; ivag, intravaginal; i.d., intradermal; MPL, monophosphoryl lipid A; HD, hydrophobic domain.

## 5. Route of Immunization

Widely used routes of delivery for HSV-2 vaccines include subcutaneous, intramuscular, intraperitoneal, intranasal, intravaginal (IVAG), intradermal, ocular and intravenous delivery. The route of immunization has an impact on the efficacy of HSV-2 vaccine via different immune responses at different sites. The different immune responses induced by immunization routes are determined by the unique nature of delivery sites. As the findings of some studies indicate, when challenged IVAG with HSV-2 MS, mice immunized with gE2-del virus by the intramuscular route all survived, in contrast with a survival rate of 60% for the mice immunized subcutaneously [75]. In addition, no mice immunized intramuscularly contracted diseases while some of the mice immunized subcutaneously developed diseases [75]. These data provide evidence that the intramuscular route is better than the subcutaneous route when live attenuated HSV-2 vaccine is administered. However, as reported in another study, when the HSV-2 DNA vaccine encoding gD2 was injected via intramuscular or footpad route in mice, intramuscular immunization induced higher levels of antibody in sera, while footpad subcutaneous immunization triggered a higher HSV-specific cytotoxicity response [82]. The reason is not fully understood why different routes of immunization lead to different types of response.

Recently, mucosal immunity has become a preferred method due to its crucial role to prevent STIs. With most STIs via mucosa such as HIV and HSV, the mucosa is the first line of defence [83]. Thus, the mucosal delivery of vaccine not only imitates the natural process of virus infection, but also induces mucosal innate immune response, which shows the potential ability to develop an optimal vaccine. Now, the mucosal route of HSV-2 vaccine immunization includes intranasal, intravaginal, ocular and oral delivery. The ocular delivery of live attenuated virus (HSV-2 ICP0^−^) induced HSV-specific IgG antibody response and protected against later virus challenge [55]. However, the ocular immunization incurs the difficulty in controlling in humans, a problem that needs to be solved in the future. In contrast, the intranasal immunization, widely used in HSV-2 vaccine research, proved to be an effective method. Another alternative, the oral delivery of the vaccine, passes through mucosa of the stomach, small intestine and large intestine. As a convenient, fast, and painless option, it is easily accepted by people, suggesting a bright future. Besides, rectal immunization of HSV-2 TK^−^ induced HSV-specific cellular and humoral immune responses as well as protection against virus challenge [84]. However, there is a debate on whether the oral delivery is better than the intranasal delivery.

## 6. Adjuvant

An optimized HSV-2 vaccine requires an appropriate adjuvant. Despite great efforts made up to now, no ideal HSV-2 vaccine adjuvant is available yet. The effective adjuvants should not only induce effective innate immune response, but also trigger an adequate adaptive immune response. The HSV-2 vaccine adjuvants commonly used in animals include alum [69,79], monophosphoryl lipid A (MPL) [70,79], cholera toxin [84], CpG-containing oligodeoxynucleotides (CpG, a TLR9 agonist) [52,67,68,69] and the double-stranded RNA analog polyinosinic-polycytidylic acid (poly (I:C), a TLR3 agonist) [29]. In recent studies, heat shock proteins (HSP) with adjuvant activity for inducing specific CD8^+^ T cell immune responses [85] have been used as HSV-2 vaccine adjuvant [86]. Of several different potent HSV-2 vaccine adjuvants studied recently, IC31, which contains the synthetic anti-microbial peptide KLK and the non-CpG motif-bearing oligodeoxynucleotide (ODN) d(IC)_13_ (ODN1a), triggered dendritic cells and induced strong antigen-specific type 1-dominated T helper cell (Th1) responses and antigen-specific CD8^+^ cytotoxic T cell response via the TLR9/MyD88-signaling pathway in mice [87,88]. The results of a subsequent study are that HSV-2 subunit vaccine containing gD2 and IC31 induced gD-specific IgG antibody in the vaginas of mice providing 80%–100% protection [89]. Polyethyleneimine (PEI), widely used as a gene transduction agent, stimulates potent mucosal adjuvant activity [90], thus increasing the immunogenicity of DNA vaccine [91,92]. However, the interaction between PEI and DNA vaccine is not clearly understood. When the mice received intranasal administration of gD2 in PEI, their antigen-specific serum IgG titer was higher than that through their receiving gD2 alone [90]. Another adjuvant is cationic liposome-DNA complexes (CLDC), which were originally developed as a system of gene delivery. It engages in the potent activity of innate immune response and inhibits gene expression [93]. Therefore, CLDC not only induced antibody and T-cell responses to HSV-2, but also decreased acute and recurrent diseases and the spread of HSV-2 in guinea pigs [93,94]. But, it failed as a therapeutic vaccine adjuvant [94]. Finally, Vaxfectin, a cationic lipid-based suspension, increased the immunogenicity of influenza pDNA vaccines in Phase 1 clinical trials [95]. Vaxfectin also improved protection provided by gD2 pDNA vaccine in both a mice model and guinea pig model [76,77]. However, whether Vaxfectin works in other types of HSV-2 vaccines should be further investigated. Besides, more research has been done on other options as HSV-2 vaccine adjuvants, inclusive of GPI-0100 (a semisynthetic Quillaja Saponin analog) [96], AS04 (a novel adjuvant system containing aluminium hydroxide and 3-d–monophosphoryl lipid) [97], β2-adrenergic agonist [98], α-galactosylceramide (αGalCer, a CD1 ligand) [99], AFCo1 (*Neisseria meningitides* B proteoliposome (AFPL1)-derived cochleate) [100] and other TLR agonists [29,101].

## 7. Influence of Sex Hormone

Female sex hormones have demonstrated their influence on acquiring STIs including HSV-2 [102,103]. Though the female sex hormones, both estrogen and progesterone, are secreted endogenously via ovaries or provided by hormonal products like hormonal contraceptives, the two types of hormones differ in their response to different variables. The mice exposed to Depo-Provera (a long-acting progestational formulation) failed to generate protection against HSV-2 following intravaginal administration of attenuated HSV-2 [104]. In the subsequent studies, the administration of Depo-Provera or a saline suspension of progesterone in mice increased the susceptibility to genital HSV-2 infections by 100-fold or 10-fold, respectively [105]. In contrast, estradiol plays a different role in protection against HSV-2. The administration of estradiol in ovariectomized mice induced protection against HSV-2 infection without showing any vaginal pathology or viral shedding [106,107], with both intranasal and subcutaneous administration of estradiol leading to the same results in a genital herpes infection model [108]. Furthermore, estradiol enhanced the efficacy of live attenuated virus vaccines [107,108]. As shown in recent studies in mice, intramuscular administration of gD2 subunit vaccine with estradiol induced higher protection by the vaccine, indicating that estradiol also enhances the efficacy of subunit vaccine [109]. However, immunity induced by rectal immunization with HSV-2 TK^−^ is via a MyD88-dependent manner rather than through a sex hormone influence [84]. Thus, more research should be conducted into the reason why progesterone increases the susceptibility of HSV-2 while estradiol increases protection against HSV-2.

The studies mentioned above clearly indicate the importance of female sex hormones in HSV-2 infection and immune response. Progesterone increases the susceptibility of HSV-2 while estradiol increases protection against HSV-2, which influences the efficacy of HSV-2 vaccine. In addition, cervicovaginal lavage (CVL) obtained from 26 women showed the potential ability to inhibit HSV infection [110]. In CVL, human neutrophil peptides 1–3, IL-8, lactoferrin, lysozyme, IgA and IgG were found to correlate with antiviral activity [110]. Also, even in the absence of secondary lymphoid organs, intravaginal administration of HSV-2 TK^−^ induced efficient memory immune responses at genital mucosa [111]. The data strongly suggest that, like the female sex hormones, the female genital tract microenvironment, also plays an important role in immune response and HSV infection, and deserves more research. Furthermore, compared with female sex hormones, the effects of male sex hormones on HSV-2 vaccine efficacy have rarely been studied, which is another research direction for the future studies.

## 8. Future Perspectives

**Figure 2:**
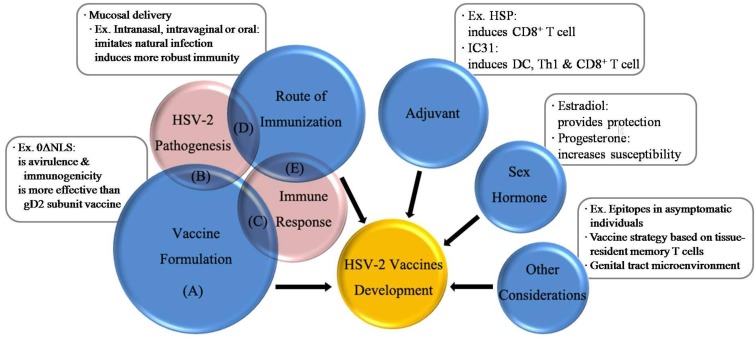
Factors of developing an effective vaccine. (**A**) The size of the circle represents the importance of the factor; (**B**) The progress in HSV-2 pathogenesis will promote the development of vaccine formulation; (**C**) The progress in HSV-2 immune response will promote the development of vaccine formulation; (**D**) The progress in HSV-2 pathogenesis will promote the development of immunization route; (**E**) The progress in HSV-2 immune response will promote the development of immunization route.

Right now there is no effective vaccine against HSV-2 available; such a situation motivates us with more enthusiasm to pursue an effective HSV-2 vaccine. An effective HSV-2 vaccine should provide multiple protection mechanisms: (a) cell immunity against viral infected epithelial cells is required; (b) humoral immunity against viral transmission and shedding is necessary; (c) innate immunity, especially mucosal immunity, is indispensable for providing long-term protection against virus invasion. Mucosal immunity can not only trigger the quick immediate innate immunity, but also induce subsequent specific adaptive immunity, holding the key for future HSV-2 vaccines. Mucosal delivery, which shows great advantages in triggering a robust immune response against the HSV-2, is another future direction. A mucosal adjuvant should be considered to enhance immunity. In addition, HSV-2 genes and proteins should be further explored, for a simple glycoprotein or several glycoproteins may not have the ability to induce robust immune responses against HSV-2. Furthermore, estradiol, which increases protection against HSV-2, can be involved in future HSV-2 vaccine development. Figure 2 illustrates a summary of the key factors with a view to providing possible strategies for future HSV-2 vaccine development
